# Supplementary figures and images for: Generation of human liver organoids from pluripotent stem cell-derived hepatic endoderms
Source: PeerJ. 2020 Oct 19;8:e9968. doi: 10.7717/peerj.9968 (PMC7580584; doi:10.7717/peerj.9968)

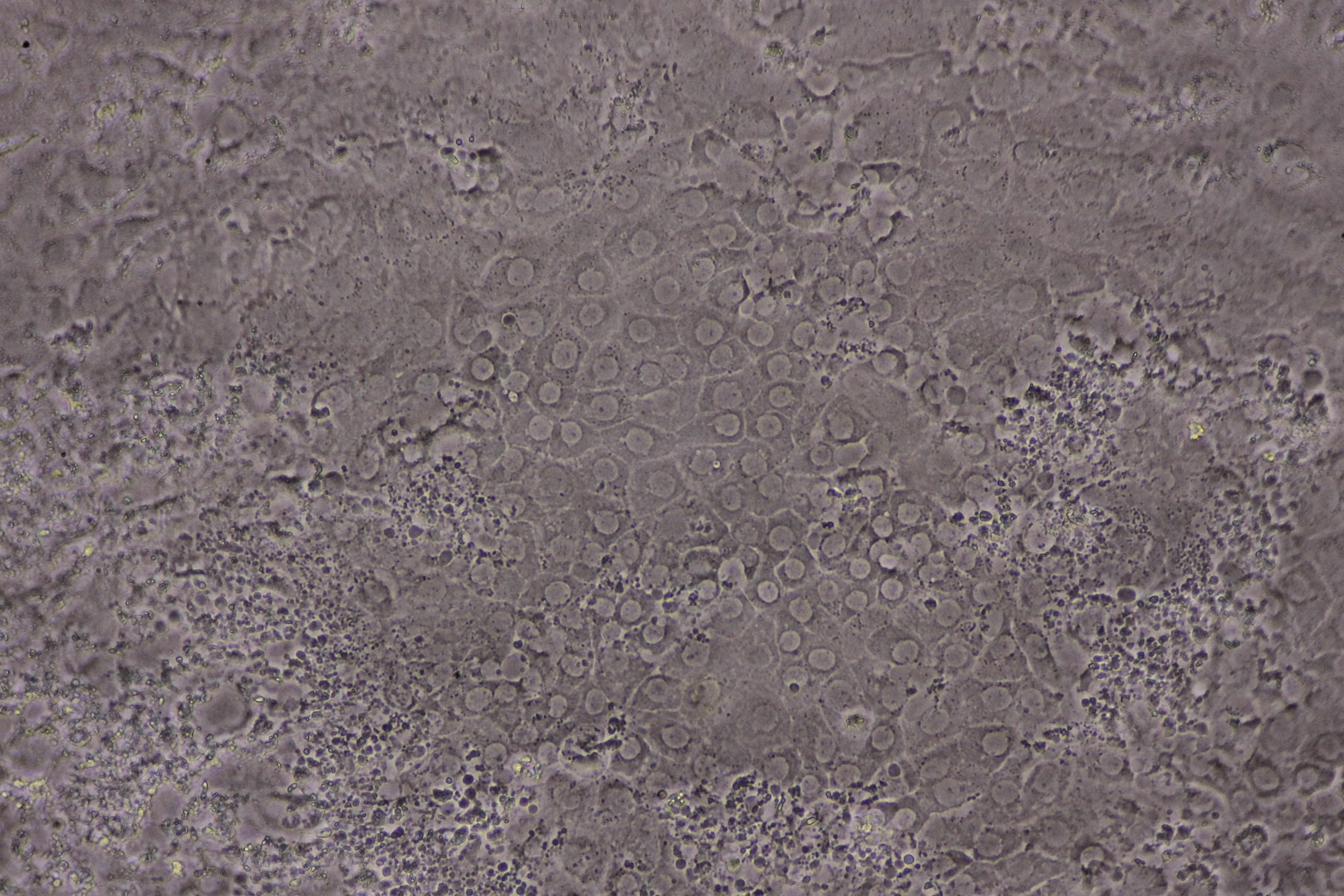

Supplement: Supplemental Information 1 — (A) Schematic diagram of hepatic cell differentiation in the 2D culture system. The protocol consisted of four stepwise phases: endoderm, hepatic endoderm, hepatoblast, and hepatocyte. Cells were cultured under 5% or 20% O2. (B–E) Representative microscopic bright-field images of differentiated iPS cells after exposure to different sets of cytokines (20X objective lens). (F) Microscopic image of 25-day-old differentiated cells at higher magnification. The dotted line indicates the area where polygonal cells having large nuclei were observed. (G–K) Gene expression profile of cells at different stages of culture. Relative expression levels were calculated using the 2ΔΔCT method. Data are the mean ± SD (n = 3) and statistically analyzed using Student’s t test. (L–P) Confocal microscopic observation of albumin (red color) and CD81 (green color). Scale bar = 50 mm. (Q) Percentage of albumin-expressing cells observed using immunofluorescence and confocal microscopy. (R) Glycogen storage in the 25-day differentiated cells from E. Representative image shown was obtained with a 100X objective lens. Light microscopic and confocal images are representative of three independent experiments. [file peerj-08-9968-s001.jpg]

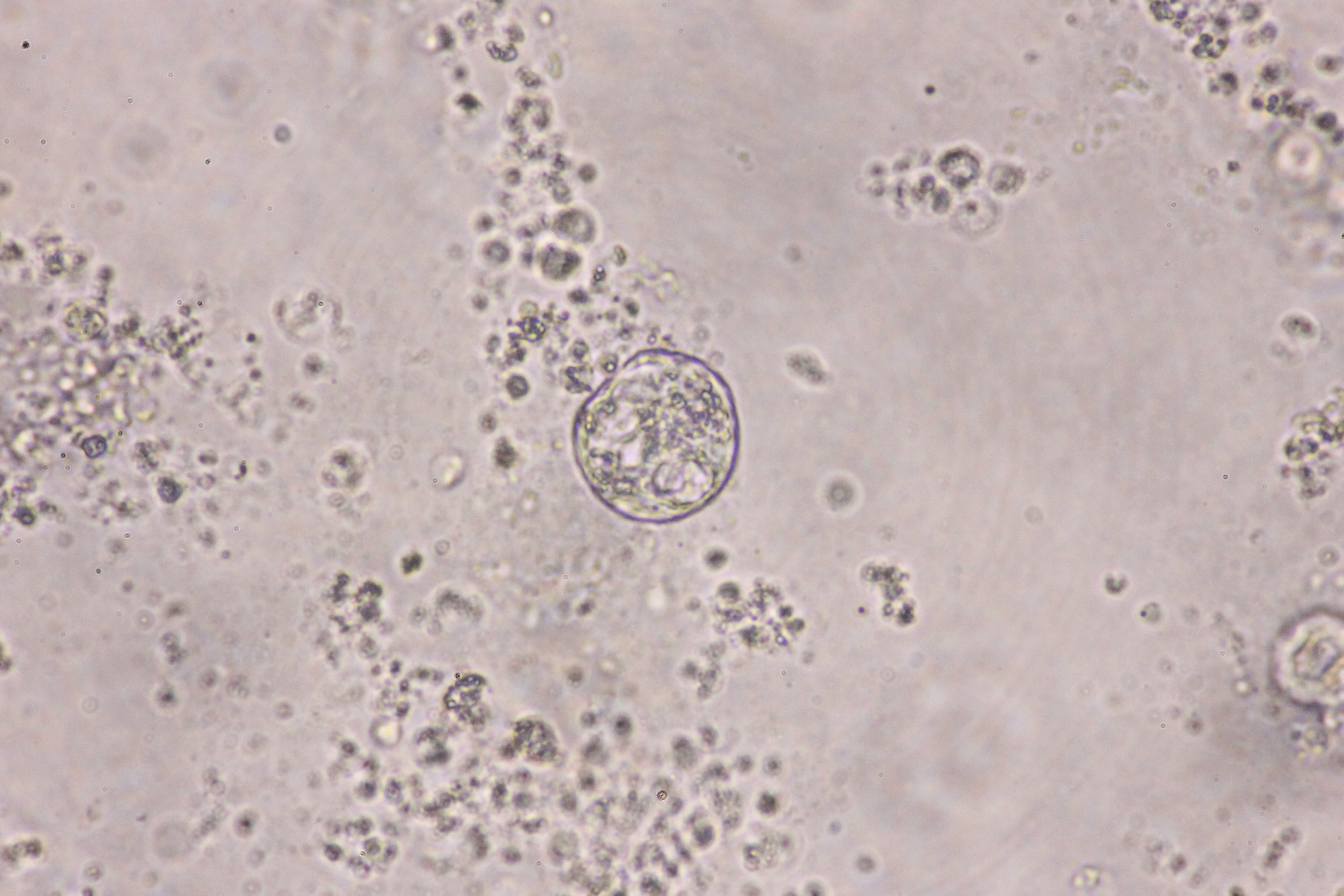

Supplement: Supplemental Information 2 — (A) Schematic diagram of the methods used for assessing organoid formation ability. (B) Hepatic endoderm (day 10), hepatoblasts (day 15), and hepatocytes (day 20) were cultured in semisolid Matrigel (pink color). Liquid medium (light orange) was added on top of the Matrigel. (C–E) Ability of hepatic endoderm, hepatoblasts, and hepatocytes to form an organoid in the Matrigel-based 3D culture was assessed at day 5 of the 3D culture. Images shown were obtained with a 40X objective lens. (F–G) Two representative images of the organoid in the Matrigel-based 3D culture at day 7. (H–I) Two representative images of the organoid in the Matrigel-based 3D culture at day 10. Three independent experiments were performed and representative microscopic images were shown. [file peerj-08-9968-s002.jpg]

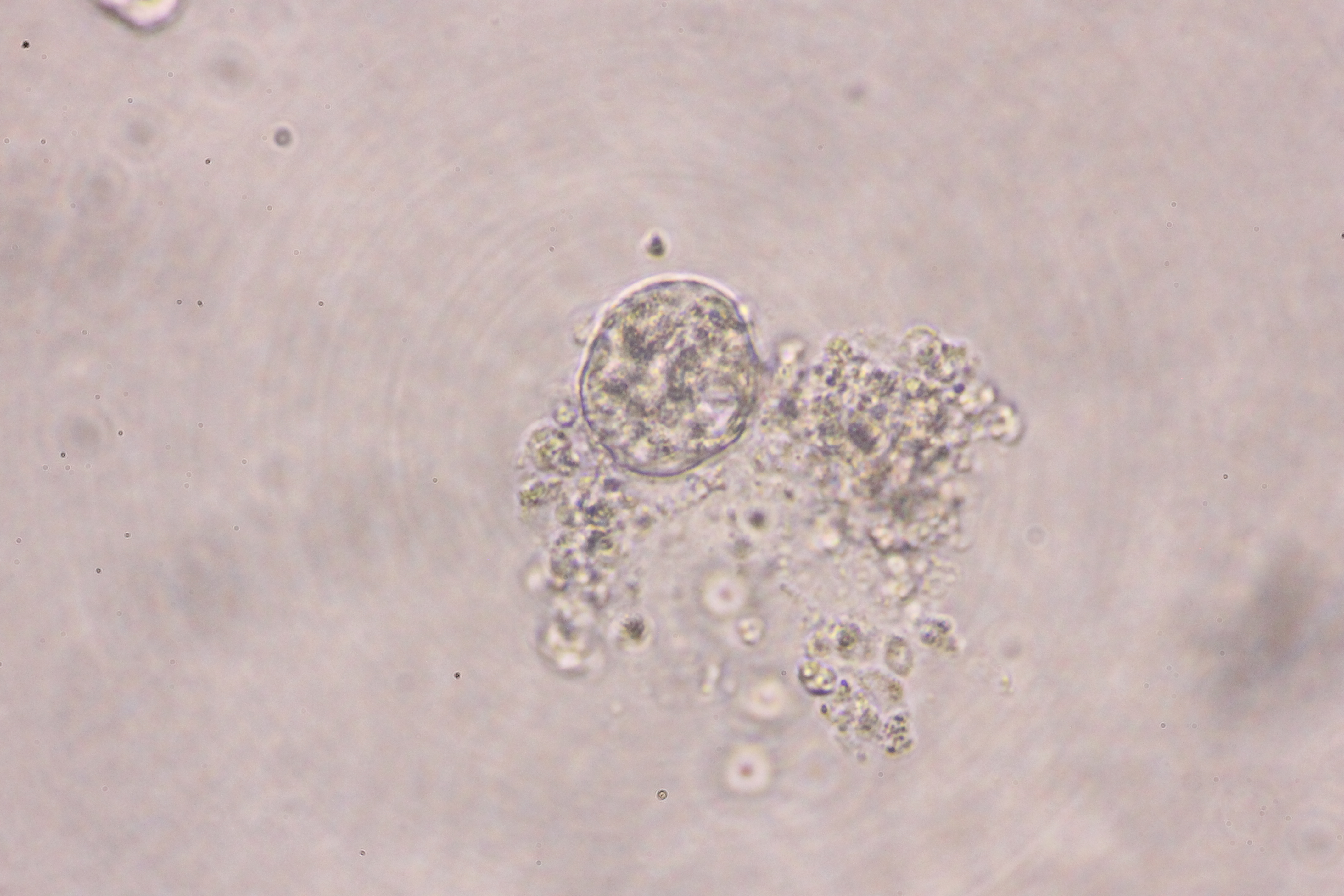

Supplement: Supplemental Information 3 — (A) Schematic diagram of the methods used for assessing organoid formation ability. (B) Hepatic endoderm (day 10), hepatoblasts (day 15), and hepatocytes (day 20) were cultured in semisolid Matrigel (pink color). Liquid medium (light orange) was added on top of the Matrigel. (C–E) Ability of hepatic endoderm, hepatoblasts, and hepatocytes to form an organoid in the Matrigel-based 3D culture was assessed at day 5 of the 3D culture. Images shown were obtained with a 40X objective lens. (F–G) Two representative images of the organoid in the Matrigel-based 3D culture at day 7. (H–I) Two representative images of the organoid in the Matrigel-based 3D culture at day 10. Three independent experiments were performed and representative microscopic images were shown. [file peerj-08-9968-s003.jpg]

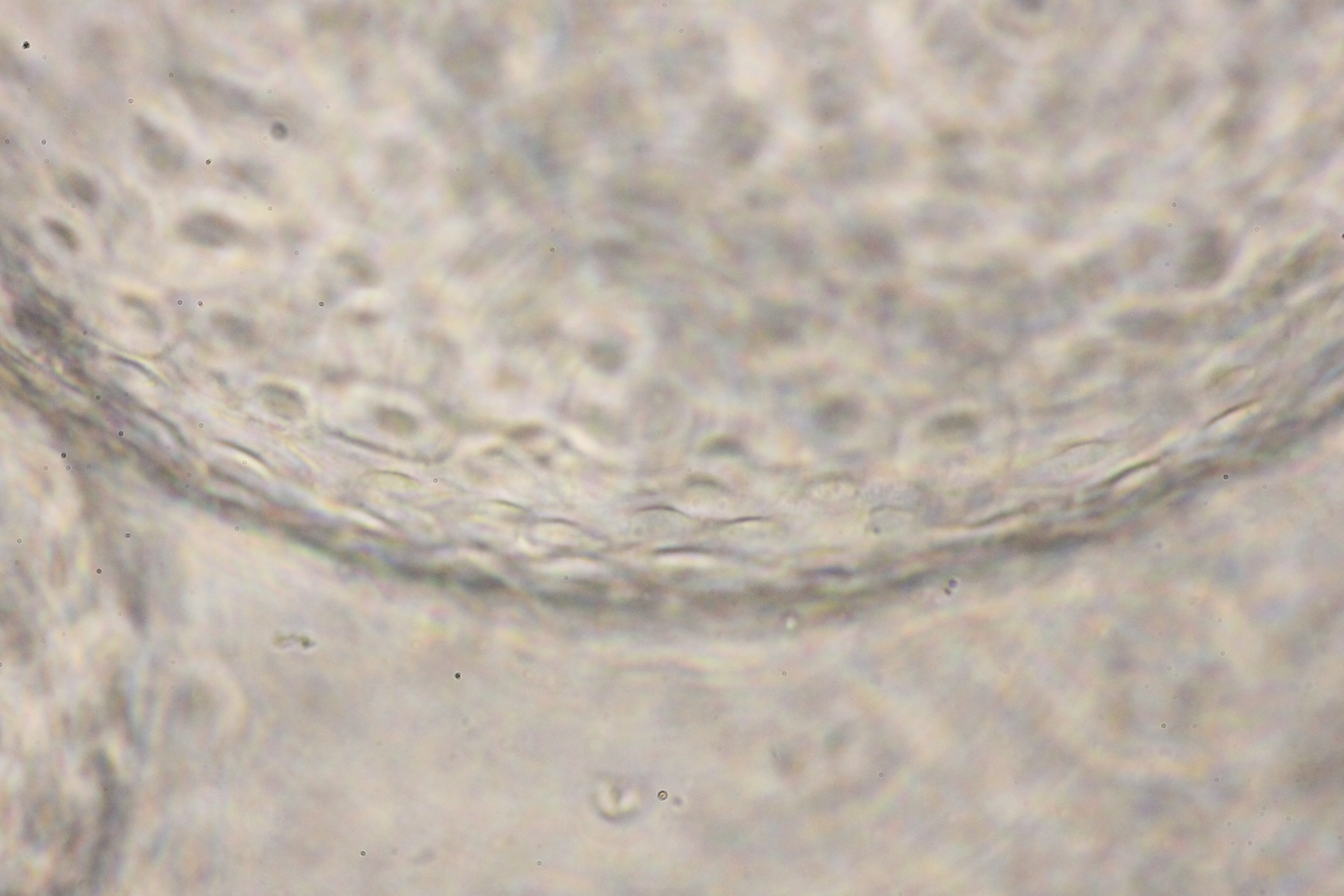

Supplement: Supplemental Information 4 — (A–C) Morphology of the HEOs at days 12, 14, and 17 postculture in Matrigel (4X objective lens). (D–F) Representative images of single HEOs are shown at high magnification (40X). (G) The outer layer of cells is shown. (H) Zoomed-in image of the HEOs showing the cells with a polygonal shape and large nuclei (arrowheads). (I–LL) Confocal images of a day 12- and 17-derived organoid showing the expression of hepatic nuclear factor 4 (HNF4), hepatocyte-specific albumin (ALB) and α-fetoprotein (AFP), cytochrome P450 3A4 (CYP4A3), and CD81, an important receptor of Plasmodium falciparum sporozoites. (MM-PP) Co-localization of CD81 and ALB in the 17-day HEO. The confocal images of organoid show the expression of CD81 (green) and hepatocyte-specific albumin (red). (QQ–TT) Zoomed-in images reveal the albumin- and CD81-expressing cells. (UU–XX) Cells stained with 2nd antibody specific to IgG of rabbit and mouse (unstained samples) served as background control. Four independent experiments were performed and representative images are shown. (YY) Total amount of human albumin secreted within 48 h in the culture medium of the 25- and 40-day 2D culture and the 17- and 60-day 3D culture. Total amount (ng) of human albumin in each experiments were calculated based on number of cells. Individuals represent independent experiments. Data are the mean ± SD (n = 3–4) and statistically analyzed using Student’s t test. Culture medium of undifferentiated HuiPSCs was used for comparison. (ZZ–EEE) Hepatocyte functions (glycogen and lipid storage, and metabolism of indocyanine) of a day 12- and 17-derived organoids. Four independent experiments were performed and representative images are shown. [file peerj-08-9968-s004.jpg]

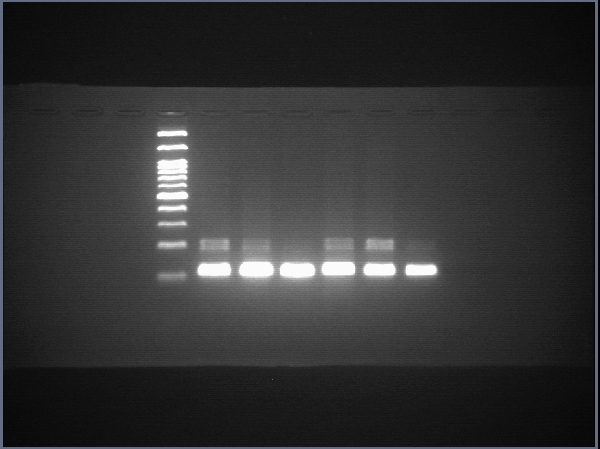

Supplement: Supplemental Information 5 [file peerj-08-9968-s005.jpg]

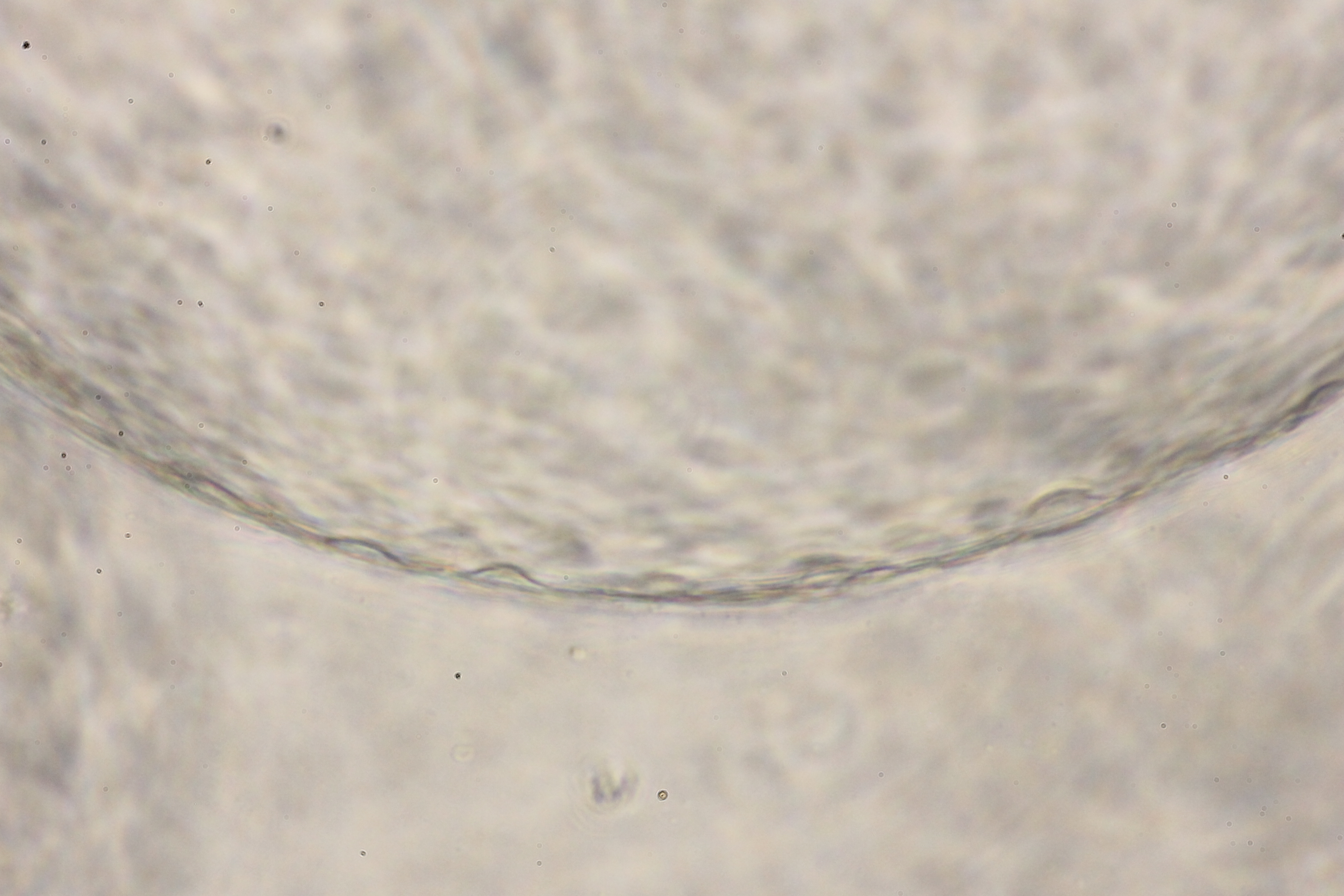

Supplement: Supplemental Information 6 — (A–C) Morphology of the HEOs at days 12, 14, and 17 postculture in Matrigel (4X objective lens). (D–F) Representative images of single HEOs are shown at high magnification (40X). (G) The outer layer of cells is shown. (H) Zoomed-in image of the HEOs showing the cells with a polygonal shape and large nuclei (arrowheads). (I–LL) Confocal images of a day 12- and 17-derived organoid showing the expression of hepatic nuclear factor 4 (HNF4), hepatocyte-specific albumin (ALB) and α-fetoprotein (AFP), cytochrome P450 3A4 (CYP4A3), and CD81, an important receptor of Plasmodium falciparum sporozoites. (MM-PP) Co-localization of CD81 and ALB in the 17-day HEO. The confocal images of organoid show the expression of CD81 (green) and hepatocyte-specific albumin (red). (QQ–TT) Zoomed-in images reveal the albumin- and CD81-expressing cells. (UU–XX) Cells stained with 2nd antibody specific to IgG of rabbit and mouse (unstained samples) served as background control. Four independent experiments were performed and representative images were shown. (YY) Total amount of human albumin secreted within 48 h in the culture medium of the 25- and 40-day 2D culture and the 17- and 60-day 3D culture. Total amount (ng) of human albumin in each experiments were calculated based on number of cells. Individuals represent independent experiments. Data are the mean ± SD (n = 3–4) and statistically analyzed using Student’s t test. Culture medium of undifferentiated HuiPSCs was used for comparison. (ZZ–EEE) Hepatocyte functions (glycogen and lipid storage, and metabolism of indocyanine) of a day 12- and 17-derived organoids. Four independent experiments were performed and representative images are shown. [file peerj-08-9968-s006.jpg]

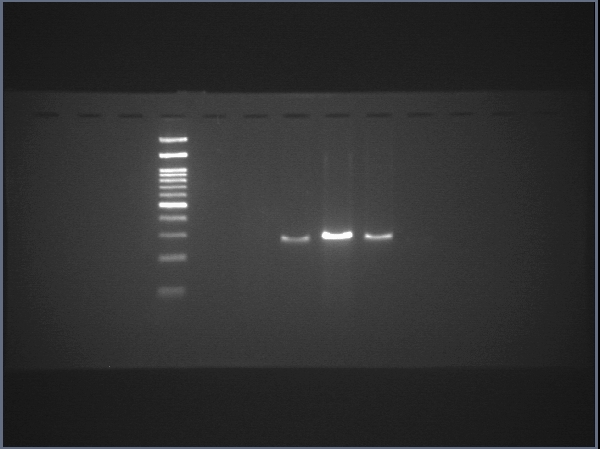

Supplement: Supplemental Information 7 [file peerj-08-9968-s007.jpg]

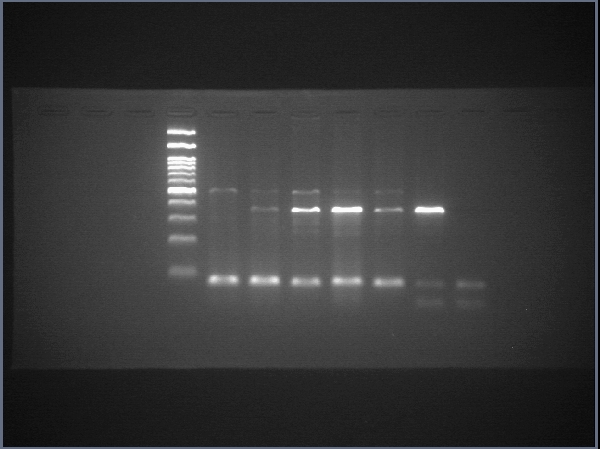

Supplement: Supplemental Information 8 [file peerj-08-9968-s008.jpg]

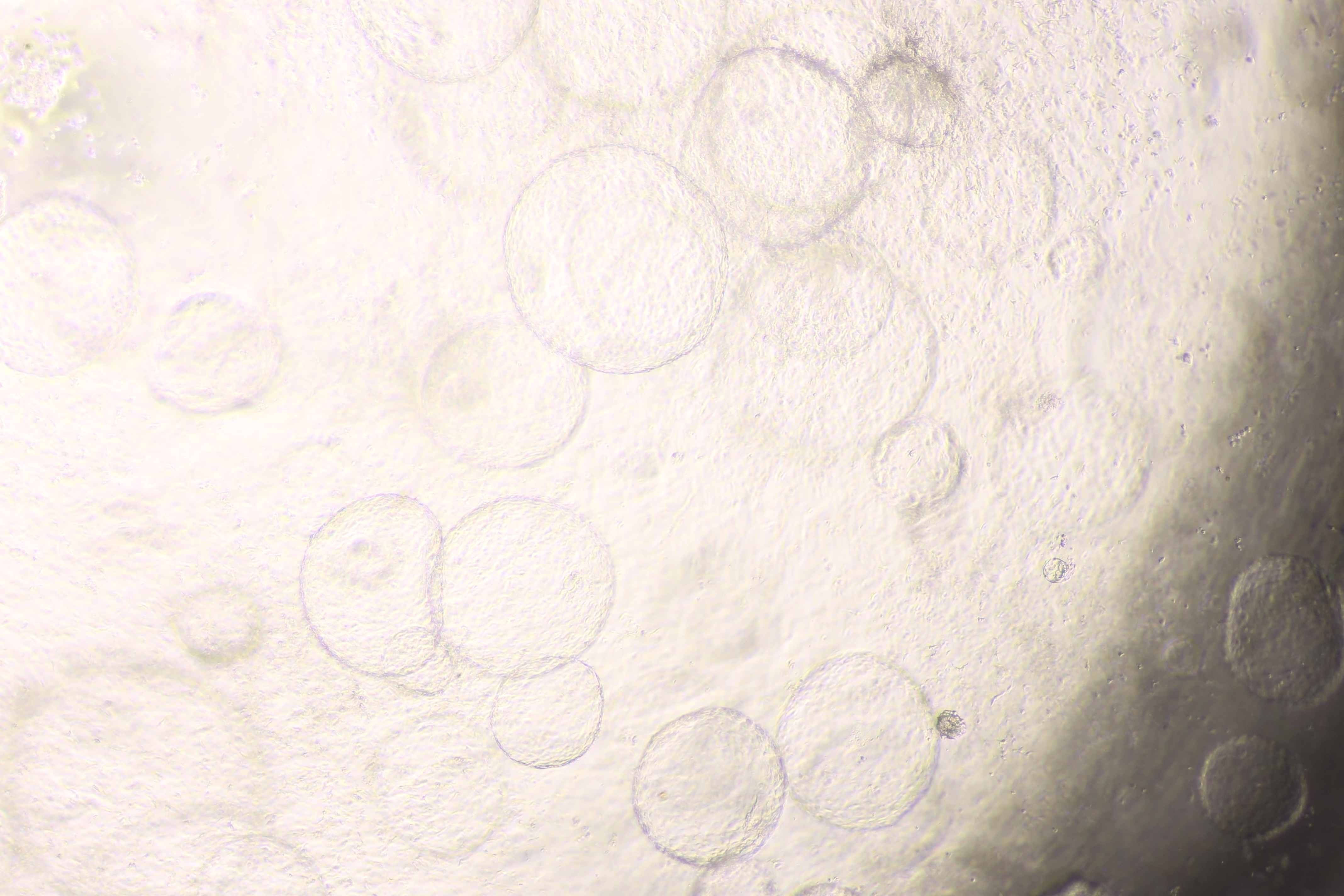

Supplement: Supplemental Information 9 — (A) Schematic diagram showing a method used to determine the minimum concentration of Matrigel in which the 3D structure of the organoid could be maintained. (B–E) Microscopic images of the liver organoid. The 20-day-old organoids were split to obtain 25%, 12.5%, and 6.25% Matrigel in RPMI/B27, which were supplemented with HGF and cultured for 5 days. All images were captured using a 20X objective lens. Three independent experiments were performed and representative images were shown. [file peerj-08-9968-s009.jpg]

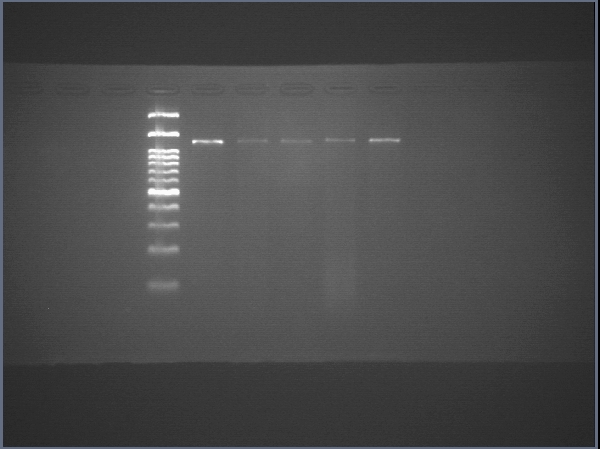

Supplement: Supplemental Information 10 [file peerj-08-9968-s010.jpg]

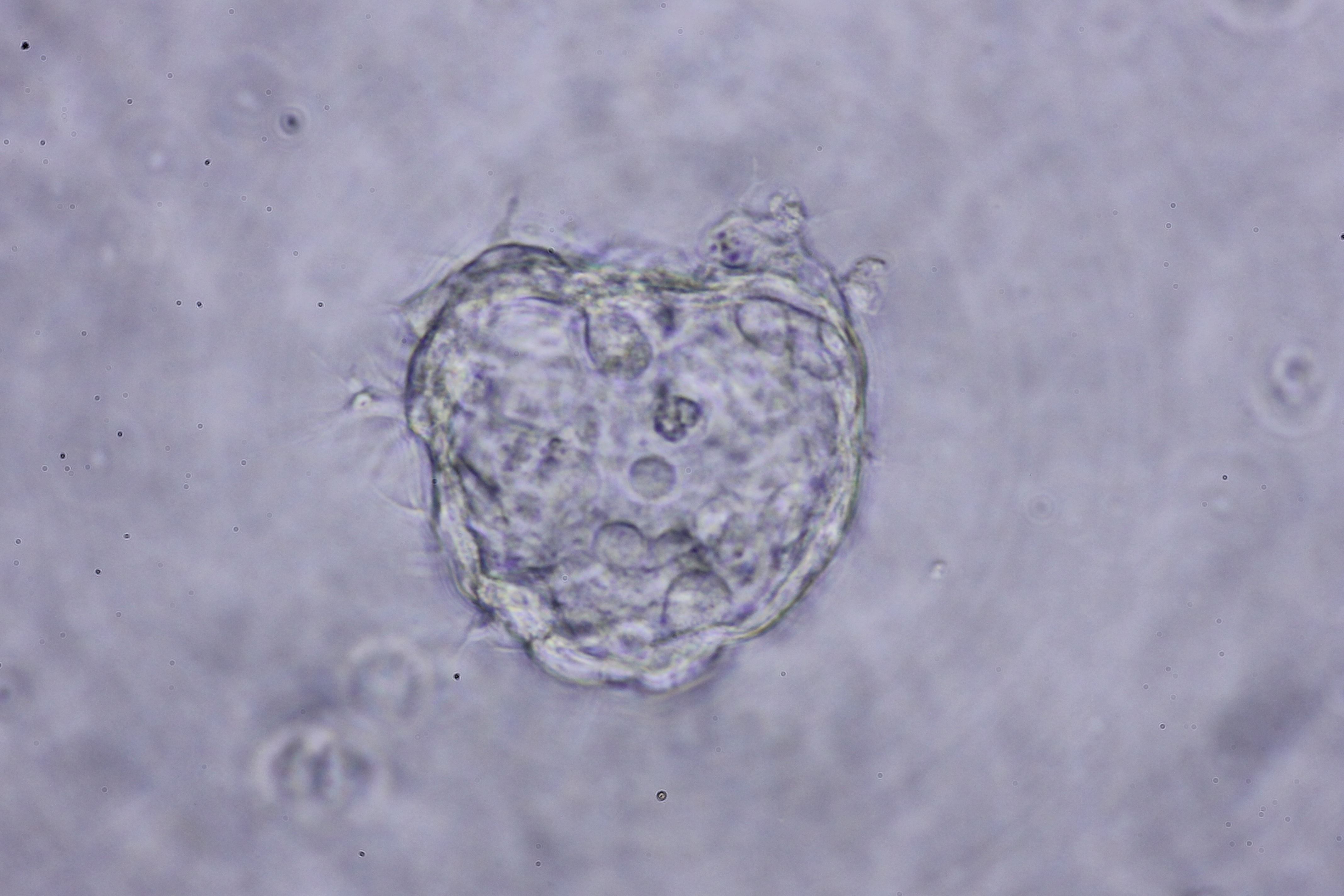

Supplement: Supplemental Information 11 — (A) Schematic diagram of the methods used for assessing organoid formation ability. (B) Hepatic endoderm (day 10), hepatoblasts (day 15), and hepatocytes (day 20) were cultured in semisolid Matrigel (pink color). Liquid medium (light orange) was added on top of the Matrigel. (C–E) Ability of hepatic endoderm, hepatoblasts, and hepatocytes to form an organoid in the Matrigel-based 3D culture was assessed at day 5 of the 3D culture. Images shown were obtained with a 40X objective lens. (F–G) Two representative images of the organoid in the Matrigel-based 3D culture at day 7. (H–I) Two representative images of the organoid in the Matrigel-based 3D culture at day 10. Three independent experiments were performed and representative microscopic images were shown. [file peerj-08-9968-s011.jpg]
