# Supplementary material for: Generation of human liver organoids from pluripotent stem cell-derived hepatic endoderms
Source: PeerJ. 2020 Oct 19;8:e9968. doi: 10.7717/peerj.9968 (PMC7580584; doi:10.7717/peerj.9968)
Supplement: Supplemental Information 12 — The uncropped images of agarose gel and the uncropped images captured under microscope. [file peerj-08-9968-s012.pptx]

## Slide 1
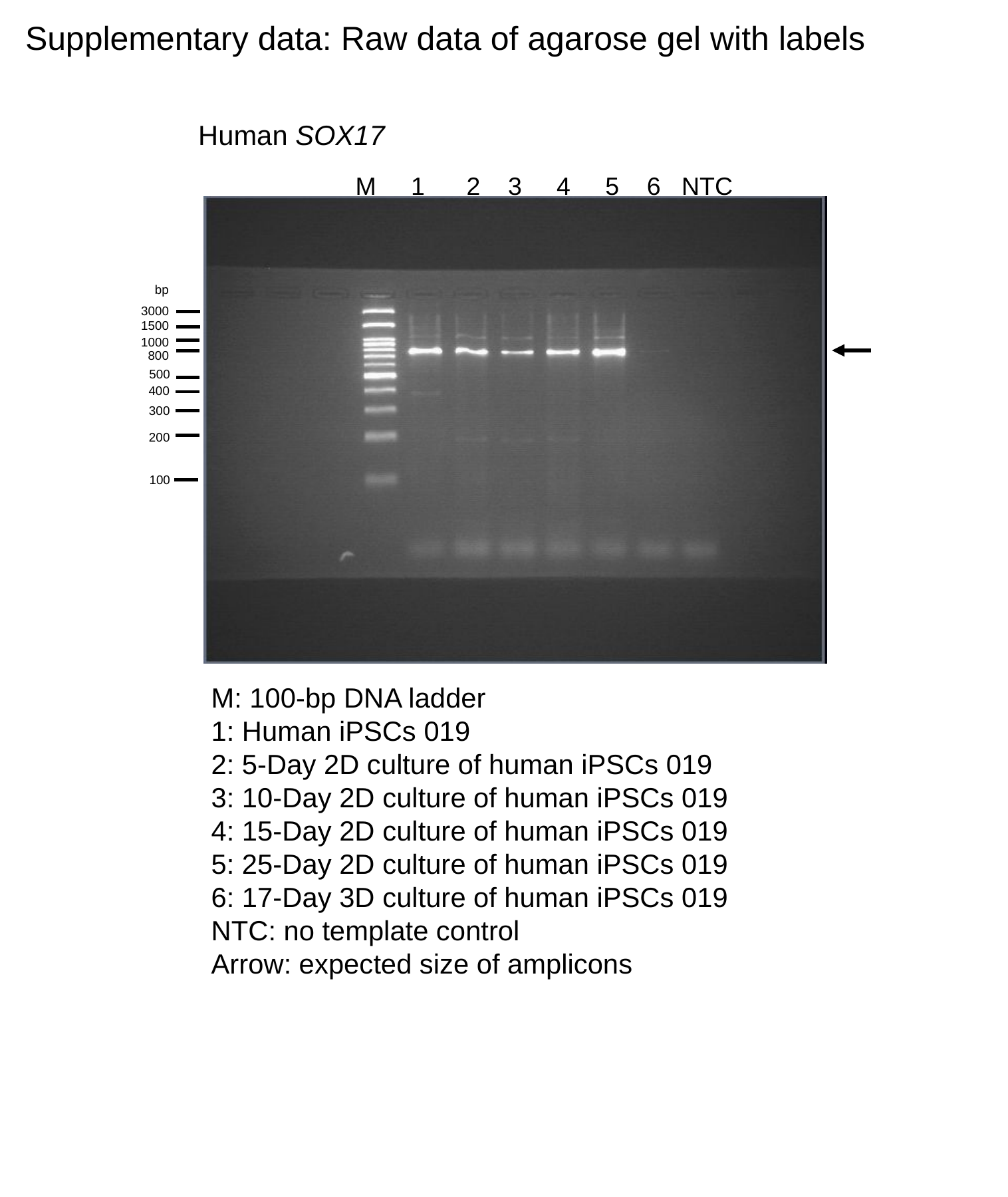

Supplementary data: Raw data of agarose gel with labels
Human SOX17
 M 1 2 3 4 5 6 NTC
bp
3000
1500
1000
800
500
400
300
200
100
M: 100-bp DNA ladder
1: Human iPSCs 019
2: 5-Day 2D culture of human iPSCs 019
3: 10-Day 2D culture of human iPSCs 019
4: 15-Day 2D culture of human iPSCs 019
5: 25-Day 2D culture of human iPSCs 019
6: 17-Day 3D culture of human iPSCs 019
NTC: no template control
Arrow: expected size of amplicons

## Slide 2
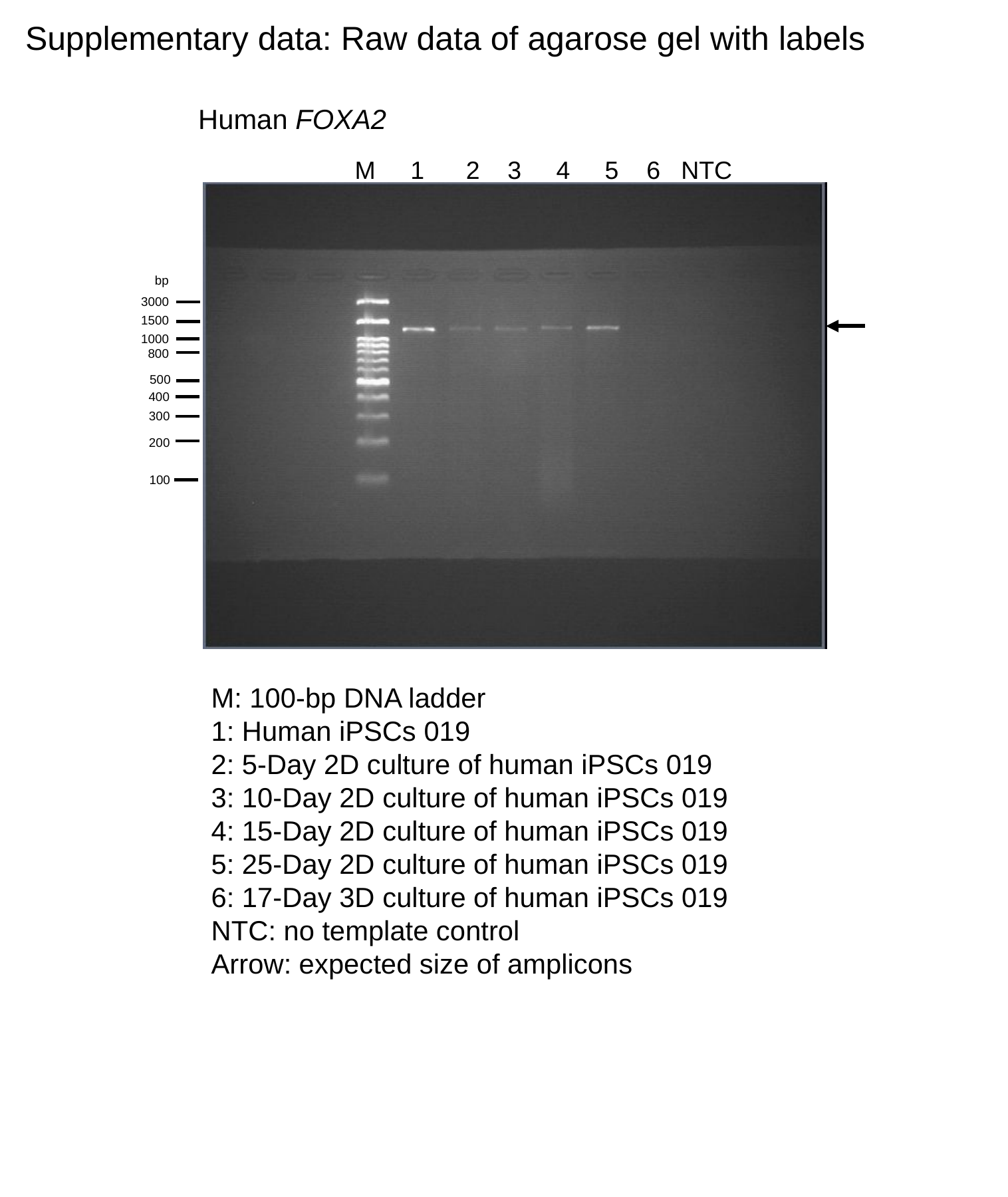

Supplementary data: Raw data of agarose gel with labels
Human FOXA2
 M 1 2 3 4 5 6 NTC
bp
3000
1500
1000
800
500
400
300
200
100
M: 100-bp DNA ladder
1: Human iPSCs 019
2: 5-Day 2D culture of human iPSCs 019
3: 10-Day 2D culture of human iPSCs 019
4: 15-Day 2D culture of human iPSCs 019
5: 25-Day 2D culture of human iPSCs 019
6: 17-Day 3D culture of human iPSCs 019
NTC: no template control
Arrow: expected size of amplicons

## Slide 3
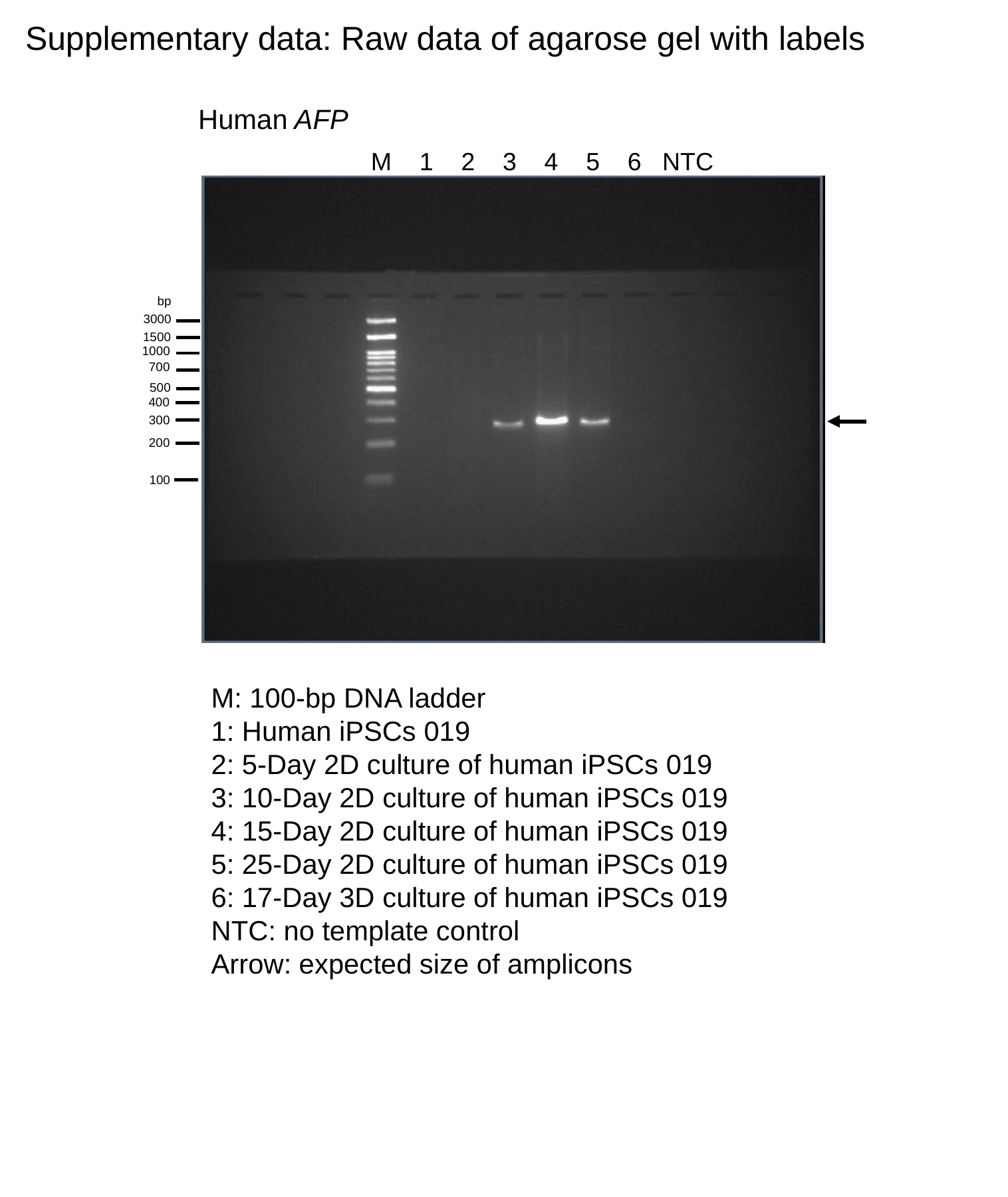

Supplementary data: Raw data of agarose gel with labels
Human AFP
 M 1 2 3 4 5 6 NTC
bp
3000
1500
1000
700
500
400
300
200
100
M: 100-bp DNA ladder
1: Human iPSCs 019
2: 5-Day 2D culture of human iPSCs 019
3: 10-Day 2D culture of human iPSCs 019
4: 15-Day 2D culture of human iPSCs 019
5: 25-Day 2D culture of human iPSCs 019
6: 17-Day 3D culture of human iPSCs 019
NTC: no template control
Arrow: expected size of amplicons

## Slide 4
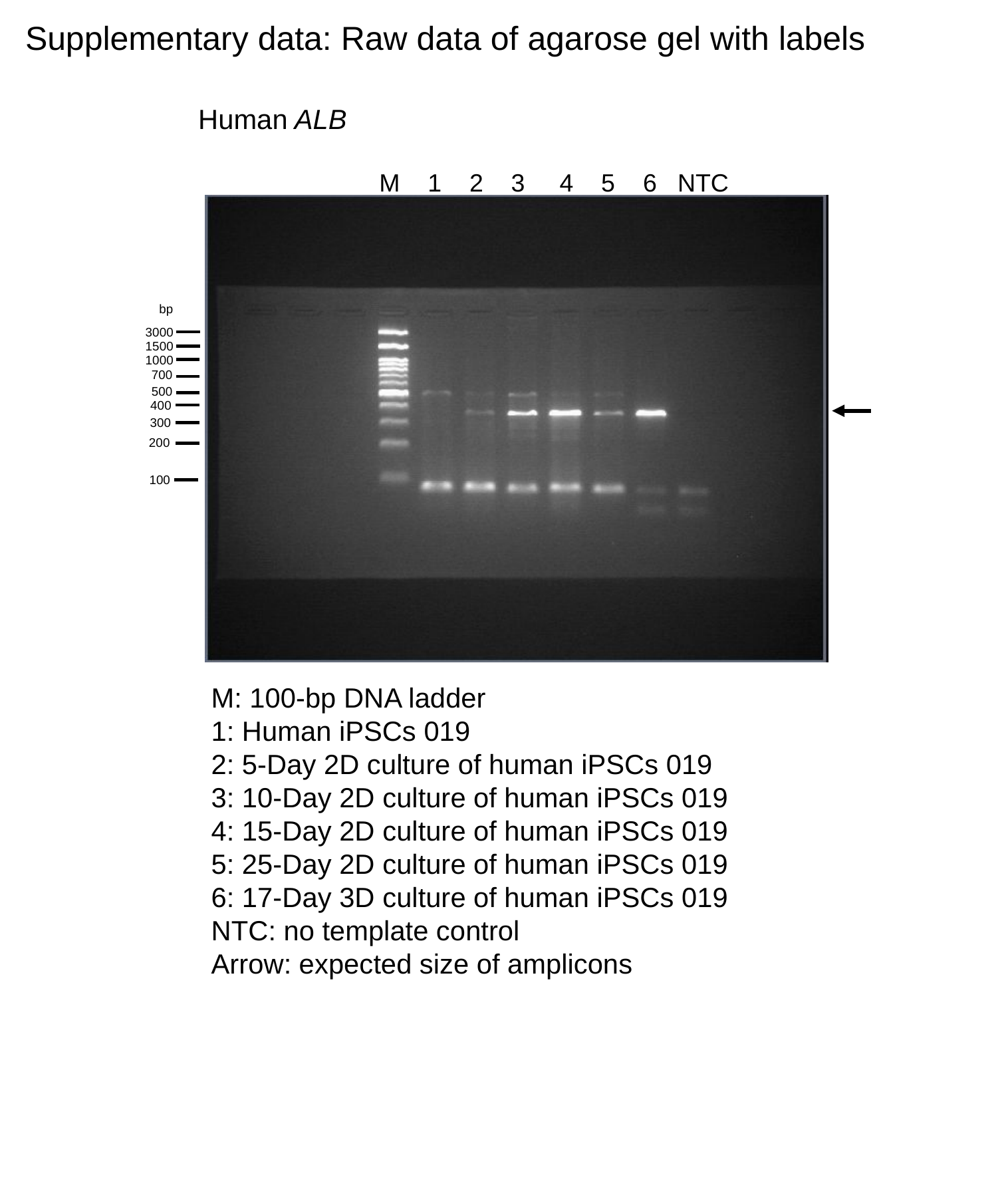

Supplementary data: Raw data of agarose gel with labels
Human ALB
 M 1 2 3 4 5 6 NTC
bp
3000
1500
1000
700
500
400
300
200
100
M: 100-bp DNA ladder
1: Human iPSCs 019
2: 5-Day 2D culture of human iPSCs 019
3: 10-Day 2D culture of human iPSCs 019
4: 15-Day 2D culture of human iPSCs 019
5: 25-Day 2D culture of human iPSCs 019
6: 17-Day 3D culture of human iPSCs 019
NTC: no template control
Arrow: expected size of amplicons

## Slide 5
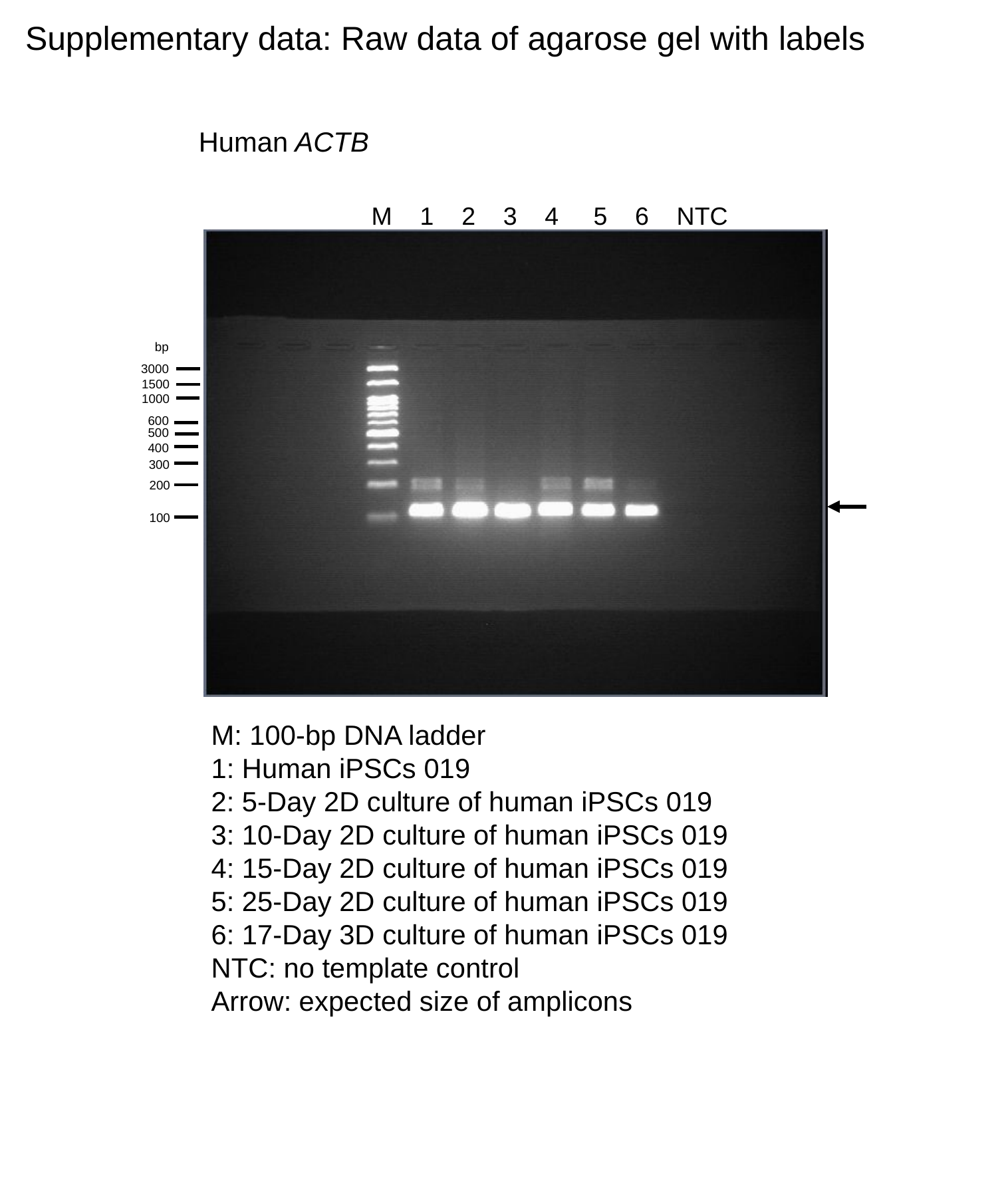

Supplementary data: Raw data of agarose gel with labels
Human ACTB
 M 1 2 3 4 5 6 NTC
bp
3000
1500
1000
600
500
400
300
200
100
M: 100-bp DNA ladder
1: Human iPSCs 019
2: 5-Day 2D culture of human iPSCs 019
3: 10-Day 2D culture of human iPSCs 019
4: 15-Day 2D culture of human iPSCs 019
5: 25-Day 2D culture of human iPSCs 019
6: 17-Day 3D culture of human iPSCs 019
NTC: no template control
Arrow: expected size of amplicons
